# Ursolic Acid induces ferroptosis by affecting redox balance and FADS2-mediated unsaturated fatty acid synthesis in Non-Small Cell Lung Cancer

**DOI:** 10.7150/jca.105863

**Published:** 2025-05-27

**Authors:** Lanlan Yang, Yuan Fang, Yuli Wang, Yingbin Luo, Yanbin Pan, Jianchun Wu, Yan Li

**Affiliations:** 1 Department of Oncology, Shanghai Municipal Hospital of Traditional Chinese Medicine, Shanghai University of Traditional Chinese Medicine, Shanghai, China; 2 Shenzhen Hospital of Guangzhou University of Traditional Chinese Medicine, Guangzhou, China

**Keywords:** Ursolic Acid, Ferroptosis, Redox Regulation, FADS2, Unsaturated Fatty Acids

## Abstract

Ursolic Acid (UA) is a naturally occurring pentacyclic triterpenoid compound that is prevalent in various medicinal plants and fruits. It has garnered significant attention due to its broad spectrum of anticancer properties. In this study, we evaluated the antitumor effects of UA on Non-Small Cell Lung Cancer (NSCLC).UA significantly inhibited NSCLC viability and induced cell death in a time- and dose-dependent manner. Furthermore, the administration of UA resulted in an elevation of intracellular reactive oxygen species (ROS), lipid ROS, and ferrous iron levels, while concurrently suppressing the expression of SLC7A11, glutathione, and GPX4. Consequently, this led to an augmentation in the concentration of the lipid peroxidation substrate, malondialdehyde. All the changes were effectively attenuated by the ferroptosis inhibitor Ferrostatin-1(Fer-1) and Deferoxamine (DFO). Moreover, similar observations were made in animal experiments. The sequencing data indicate that UA influences ferroptosis by modulating Fatty Acid Desaturase-2 (FADS2). The reintroduction of FADS2 through ectopic expression restored the resistance to ferroptosis induced by UA in A549 cells, while the addition of exogenous oleic acid (OA) counteracted the impact of UA on the oxidative response. These results suggest that UA induces ferroptosis in NSCLC by affecting redox pathways and the FADS2-mediated synthesis of unsaturated fatty acids.These studies collectively underscore the promising role of UA in the development of effective anticancer therapies.

## Introduction

Lung cancer represents a significant challenge within the realm of malignant tumors. According to the World Health Organization's 2020 statistics, the global incidence of lung cancer reached 2.2 million cases, ranking it second among malignant tumors, with a corresponding 1.8 million deaths attributed to lung cancer 1. Although various methods such as surgery, radiotherapy, and chemotherapy can be used to treat lung cancer nowadays, new drugs and methods are still necessary to target lung cancer treatment and prevention of recurrence and metastasis.

Ferroptosis was first proposed by Dixon in 2012 as a new mode of cell death, which is an iron-dependent, regulated cell death driven by lipid peroxidation that has received much attention in recent years 2. Ferroptosis has already been validated to be an effective tumor therapeutic strategy: it is shown that a variety of oncogenes or oncogenic signals can act by regulating ferroptosis, for example, the ferroptosis activator Erastin selectively kills RAS mutant tumor cells 3; several cancer cells with specific metabolic traits, such as lipid-enriched 4, ferruginous cells 5, and specific mutations (e.g Kras, TP53 mutations 6) are susceptible to ferroptosis; moreover, drug-resistant tumor cells 7 and cells with high mesenchymal stroma exhibit a higher sensitivity to ferroptosis 8,9. The therapeutic potential of ferroptosis in aspects related to tumor cell resistance, EMT, and immunosuppression has given a new spark to targeting ferroptosis for lung cancer treatment 10. Recent studies have shown that dysregulation of lipid metabolism is closely related to cancer progression and drug resistance 11-13. Since ferroptosis is characterized by the accumulation of lethal lipid peroxides through the peroxidation of phospholipids containing polyunsaturated fatty acids (PUFAs), ferroptosis and lipid metabolism are inextricably linked, regulating ferroptosis and thus suppressing tumors by affecting lipid metabolism may become a new therapeutic approach.

Previous studies have demonstrated the diverse anticancer properties of Pentacyclic triterpenoids 14, with reported effectiveness in gastric cancer, colorectal cancer, bladder cancer, lymphoma, and other malignancies 15-17. UA, a specific type of Pentacyclic triterpenoids, exhibits notable anticancer activity and has been identified as a potential therapeutic agent for anticancer treatment. Several articles have indicated that UA possesses the ability to impede cancer cell proliferation, regulate Immunity, induce apoptosis, and more 18-21. There have been reports in gastric cancer 22, colon cancer 20, and leukaemia 23 that UA inhibits tumors by inducing ferroptosis in tumor cells. Nevertheless, the specific mechanism by which UA induces ferroptosis in lung cancer remains undetermined and unexplained. Our study successfully elucidated the phenomenon and mechanism of UA-induced ferroptosis in lung cancer, specifically through the modulation of the FADS2-mediated unsaturated fatty acid synthesis suggesting that UA holds potential as a therapeutic agent for NSCLC, and providing a novel approach for the treatment of NSCLC.

## Materials and Methods

### Cell culture and death detection

A549, NCI-H1299, CALU-1, LLC, Besa-2B cell lines were obtained from the American Tissue Culture Collection (ATCC). A549, CALU-1, LLC, Besa-2B were cultured in high glucose DMEM medium (Gibco), and H1299 were cultured in RPMI-1640 medium (Gibco). All solution contains 10% fetal bovine serum (BI). In an atmosphere containing 5% CO_2_, cells were maintained at 37°C.Only cells in the exponential growth phase, confirmed to be free of mycoplasma contamination, were used for subsequent experiments to ensure result validity.

We seeded cells into 96-well plates according to 5000/well and placed them in a cell culture incubator for 24 h. We added medium containing UA (HY-N0140, MCE) or in combination with Ferrostatin-1(HY-100579, MCE), Deferoxamine(HY-B1625, MCE), Z-VAD-FMK(S7023, Selleck), Necrostatin-1(HY-15760, MCE), Bafilomycin-A1 (HY-100558, MCE) were added for 24h, and then add the medium containing CCK8 (CK-04, Dojindo) reagents and measure the absorbances byenzyme labeling instrument (Bio Tek, USA) instrument at 450 nm (Drug and inhibitor dissolution followed the instructions provided by the manufacturer, with concentrations determined based on relevant literature search).

### SYTOX Green fluorescence observation

A549 and H1299 cells were inoculated at a density of 20,000 cells/well into 48-well plates and incubated overnight at 37°C in an incubator. Following 24 hours of drug intervention, the plates were washed twice with PBS. Subsequently, culture medium containing 100 nM SYTOX Green (S7020, Invitrogen) was added. The plates were then incubated for 30 minutes in the absence of light and recorded using a fluorescence microscope (Leica, Germany) with an excitation wavelength of 488 nm.

### Intracellular ROS detection

A549 and H1299 cells were seeded in 12-well plates at a density of 50,000 cells per well. The cells were incubated for 24 hours and subsequently treated with UA or UA in combination with Fer-1 at a concentration of 1 μM. After 24 hours, the A549 and H1299 cells were washed three times with PBS, detached using pancreatin without EDTA, and resuspended in 1 µM DCFH-DA (S0033M, Beyotime) solution in PBS. The cells were then cultured in a humidified incubator at 37°C for 1 hour. The ROS levels were measured using a BD FACS Canto II flow cytometer (BD Biosciences).

### Lipid ROS observation and detection

A549 and H1299 cells were seeded in confocal dishes at a density of 2,0000 cells. On the second day, treat them with UA or Fer-1 interventions. Following a 24-hour incubation period, the cells were washed three times with PBS and examined under a confocal microscope to capture images. In short, the flow cytometry assay for C11-Bodipy was conducted similarly to that of DCFH-DA. A549 and H1299 cells (50,000/well) were plated before and treated with UA, Fer-1or Oleic Acid (ST2053, Beyotime). After 24 hours, washed with PBS, then were shed using pancreatin without EDTA, and resuspended by 1 µM C11-Bodipy (D3861, Invitrogen), in PBS. Cultured them in a humidified incubator at 37°C for 1 hour and assayed by BD FACSCanto II flow cytometer.

### Measurement of IntraCellular glutathione (GSH) and malondialdehyde (MDA)

A549 and H1299 cells were plated and underwent drug intervention, then tested as required by the Glutathione Kit (BC1175, Solarbio) and the MDA Assay Kit (BC0025, Solarbio). Animal blood samples were centrifuged at 3000rpm15 min to obtain serum and tested according to the kit protocol.

### Detection of ferrous levels

A549 and H1299 cells were seeded and exposed to the UA and DFO treatments for 24 hours. FerroOrange (Dojindo, 1μM dissolved in PBS) was added to the cells, and incubated in a cell culture incubator for 30 min. After washing the excess probes with PBS, cells were collected and processed for flow detection.

### RNA extraction and library construction

RNA was isolated and purified from each sample using TriIzol reagent, and the amount and purity of total RNA were assessed using NanoDrop. Subsequently, mRNA was purified using polyadenylate selection with oligo(dT) magnetic beads. Reverse transcription, synthesis of double-stranded cDNA, end repair, and PCR amplification were then carried out. The average insert size for the final cDNA library was 300±50 bp. Finally, we performed the 2×150bp paired-end sequencing (PE150) on an illumina Novaseq™ 6000(LC-Bio Technology CO., Ltd., Hangzhou, China) following the vendor's recommended protocol.

### Bioinformatics analysis of RNA-seq

After the final transcriptome was generated, StringTie (https://ccb.jhu.edu/software/ stringtie) and was used to estimate the expression levels of all transcripts. StringTie was used to perform expression level for mRNAs by calculating FPKM. The differentially expressed mRNAs were selected with |log2 (fold change, FC)|>1 and *p* value < 0.05 by R package edgeR(https://bioconductor.org/packages/release/bioc/html/edgeR.html). Gene ontology (GO) functional enrichment and Kyoto Encyclopedia of Genes and Genomes (KEGG) pathway enrichment were performed using the DAVID database and visualized by the ggplot package in R software. Gene set enrichment analysis (GSEA) was based on the C5 subset retrieved from the Molecular Signature Database.

### Lentivirus transfection

In our study, human FADS2 overexpression lentiviruses (FADS2-OE, HBLV-h-FADS2-3xflag-ZsGreen-PURO) and an empty vector lentivirus (FADS2-EV, HBLV-ZsGreen-PURO) were purchased from HENBIO (Shanghai, China). A549 cells were stably infected with the empty vector and the FADS2 overexpression lentiviruses for all subsequent experiments.

### Western blot analysis

Incorporate diverse cell or animal tissue samples subjected to different treatments into RIPA lysis buffer, followed by low-temperature, high-speed centrifugation. Then, add loading buffer and boil to denature the samples. Cell lysates were separated by SDS-PAGE, transferred to PVDF membranes, and incubated with primary antibodies overnight. Following washing steps, the membranes were incubated with HRP-conjugated secondary antibodies and visualized using enhanced chemiluminescence. Antibodies for the experiments used were anti-GAPDH (5174, CST), anti-GPX4 (67763-1, proteintech), anti-SLCA711(BM5318, BOSTER), anti-SCD (2794S, CST), anti-FADS2 (68026-1, proteintech).

### *In vivo* xenograft studies

For the subcutaneous tumor model, we injected A549 cells (5×10^6 cells/100µl PBS) subcutaneously into the dorsal subcutaneous left side of nude mice. After injection, The short diameter (a) and the long diameter (b) of the tumor were recorded with a vernier caliper to calculate the tumor volume (V) as V = (a^2 * b)/2, and when the tumor volume reached about 200 mm, the mice were randomly divided into 3 groups to start the intervention and measure weight every other day:1. Control group (intraperitoneal injection of 25 mg/kg/day saline), 2. UA treatment group (intraperitoneal injection of 25 mg/kg/day), 3. UA combined with Liproxstatin-1(intraperitoneal injection of 25 mg/kg/day UA and 10 mg/kg/day Lip-1). At the end of the study, the tumors were taken for tumor weight measurement, followed by immunohistochemical staining, westerrn blot, and metabolic indexes.

### Immunohistochemistry

For the tumor tissues obtained from animal experiments, three tumor samples were collected from each of the three experimental groups. The tumors were fixed in 4% paraformaldehyde, followed by dehydration using alcohol at varying concentrations. Subsequently, the tissues underwent a transparency treatment with xylene, were embedded in paraffin, and sectioned. Antigen retrieval was performed using Citrate Antigen Retrieval Solution (P0081, Beyotime). The sections were then treated with 0.3% hydrogen peroxide for 20 minutes to inhibit endogenous peroxidase activity. Subsequently, the sections were incubated overnight at 4°C with 2% bovine serum albumin and primary antibodies, including Ki67 (1:200, ab16667, Abcam), GPX4 (1:200), and FADS2 (1:200). The following day, the sections were incubated with horseradish peroxidase-conjugated secondary antibodies according to the manufacturer's instructions. Finally, the sections were stained with hematoxylin. All sections were scanned using a digital pathology scanning system (Leica). ImageJ software was employed for the quantification of the positive area and subsequent statistical analysis.

### Statistical analysis

The data values were expressed as the mean ± standard deviation of three independent trials. Statistical analyses were conducted using GraphPad Prism8 software, employing one-way analysis of variance (ANOVA) and two-tailed unpaired Student's t-tests (p < 0.05*, p < 0.01**, p < 0.001***, or p < 0.0001^#^). Flow cytometry data were analyzed using FlowJo 10.4.0 software, whereas western blot images were analyzed using ImageJ 1.53 software.

## Results

### UA-induced NSCLC cell death can be rescued by ferroptosis inhibitors

**Figure 1:**
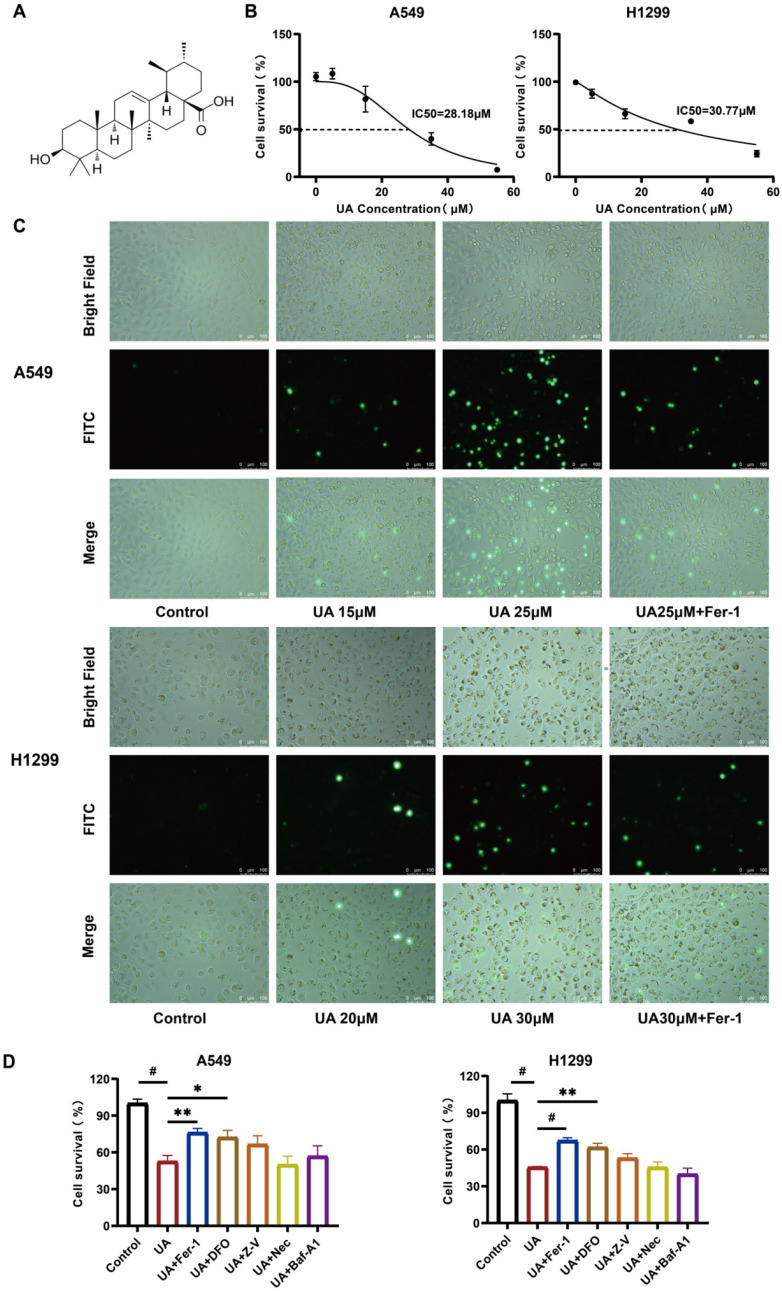
UA suppresses NSCLC growth by inducing ferroptosis. (A)Chemical structure of ursolic acid (UA). (B)The IC50 of A549 and H1299 was determined by the CCK-8 method after 24h of UA treatment. (C)A549/H1299 cells were observed at different concentrations of UA and combined with Fer-1 for 24h, SYTOX Green (100 nM) assay was performed by fluorescence microscopy. Scale bar:100μm. (D) A549/H1299 were treated with UA alone and in combination with Fer-1(1μM), DFO (60μM), Z-V(20μM), Nec(20μM), Baf-A1(50nM) for 24h, then the cell viability was detected by CCK8 assays.

To determine the drug action concentration, we initially examined the effect of UA on A549, H1299, CALU-1, LLC, Besa-2B cells. The chemical structure of UA was displayed in Figure 1A. After treating the cells with UA for 24 hours, we examined them by cck8, and the results showed that UA could induce a dose-dependent decrease of cell viability in Lung Cancer cells, but exhibited less cytotoxicity in lung epithelial-derived cells (Figure 1B, Figure S1). The half-maximal inhibitory concentration values (IC50) were 28.18μM and 30.77μM (Figure 1B). We selected the appropriate concentration of UA combined with Fer-1 and acted on A549 and H1299 cells after 24 hours, the cell death was exposed to SYTOX dye, which could be visually observed under a fluorescence microscope (Figure 1C). The results demonstrated that both cells showed an increase in damage with increasing concentration of UA. While cell death was inhibited in the UA combined with the Fer-1 group compared with the high concentration UA group, suggesting that the ferroptosis inhibitor protected cancer cells against UA. Next, we applied UA and UA in combination with Ferrostatin-1, Deferoxamine, Z-VAD-FMK, Necrostatin-1, and Bafilomycin-A1 in A549 and H1299 cells (Figure 1D). Ferrostatin-1 is a ferroptosis inhibitor that inhibits Erastin-induced cellular ferroptosis. Deferoxamine is an iron chelator that binds ferric ions, thereby inhibiting ferroptosis. Z-VAD-FMK acts as a pan-caspase inhibitor. Necrostatin-1 is a necroptosis inhibitor targeting RIP1. Bafilomycin-A1 is a reversible and selective inhibitor of V-ATPases, which inhibits autophagosome-lysosome fusion, thereby inhibiting late autophagy. The results suggested that the combination of Fer-1 and DFO partially rescued the cell viability of A549 and H1299, and the difference was statistically significant. Our data showed that cotreatment with ferroptosis inhibitors, reduced UA-induced cell death, indicating that ferroptosis contributes to UA-induced cell death in NSCLC.

### UA disrupted Redox Homeostasis via the SLC7A11/GSH/GPX4 Axis

**Figure 3:**
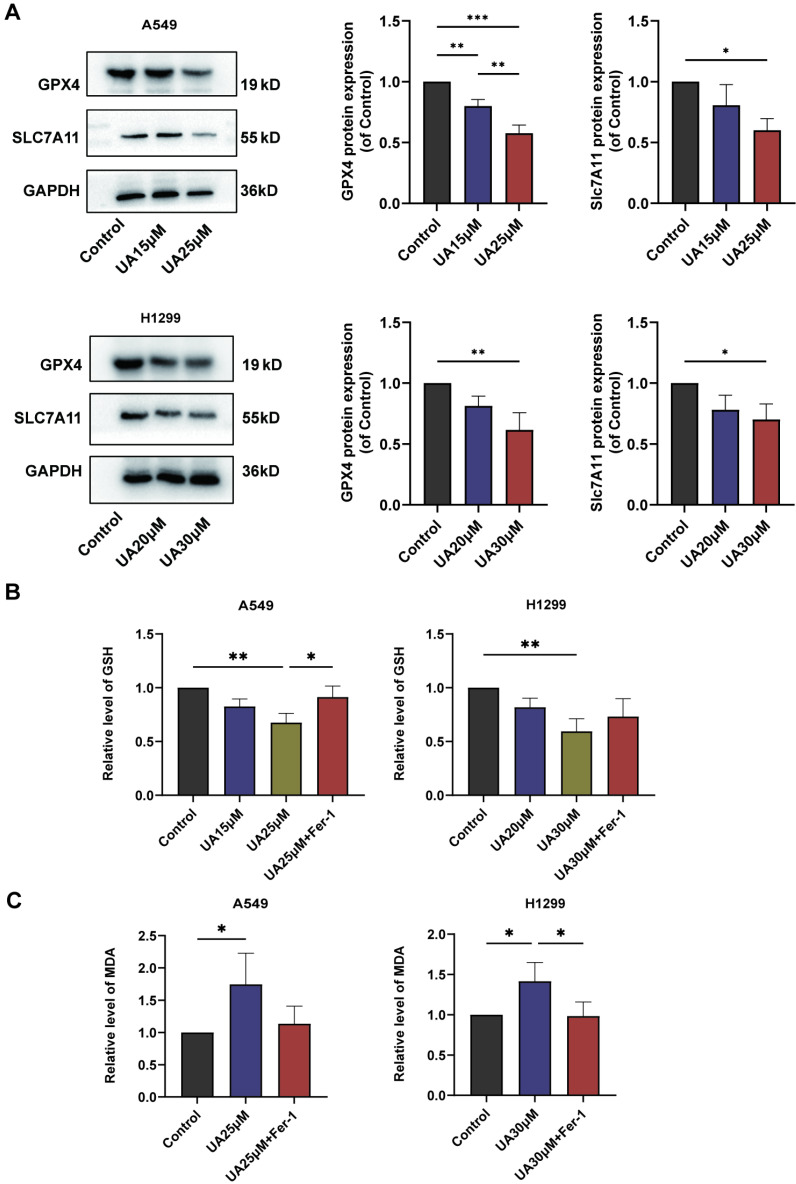
UA disrupted Redox Homeostasis. (A) The expression of SLC7A11 and GPX4 in A549\H1299 was analyzed by western blot analysis (GAPDH as an internal control). (B) Relative changes of GSH in A549\H1299 were treated with different concentrations of UA and combined with Fer-1 for 24h. (C) Relative changes of MDA in A549\H1299 exposed to UA and UA combined with Fer-1 for 48h.

Ferroptosis is frequently accompanied by a redox imbalance. We assessed the alterations in intracellular redox imbalance through GSH assay, GPX4 and SLC7A11 protein expression analysis, and MDA assay. Initially, we investigated whether both A549 and H1299 cell lines exhibited decreased GSH levels after exposure to different concentrations of UA for 24 hours compared to the control group. However, the addition of Fer-1 partially mitigated this reduction in GSH content (Figure 3B). Subsequently, western blot experiments were conducted to validate changes in GPX4 and SLC7A11 protein expression levels. The results demonstrated that protein expression declined with increasing UA concentration gradient (Figure 3A). MDA, a prominent product of lipid peroxidative damage, was observed to be significantly increased in UA-treated cells after 48 hours compared to the control group. Furthermore, treatment with Fer-1 partially attenuated this elevation (Figure 3C). Collectively, these findings indicated that UA disrupts NSCLC homeostasis while also impairing the SLC7A11/GSH/GPX4 antioxidant system, thereby creating favorable conditions for ferroptosis.

### mRNA sequence analyses indicated that UA-induced ferroptosis in NSCLC through the lipid metabolic pathway

**Figure 4:**
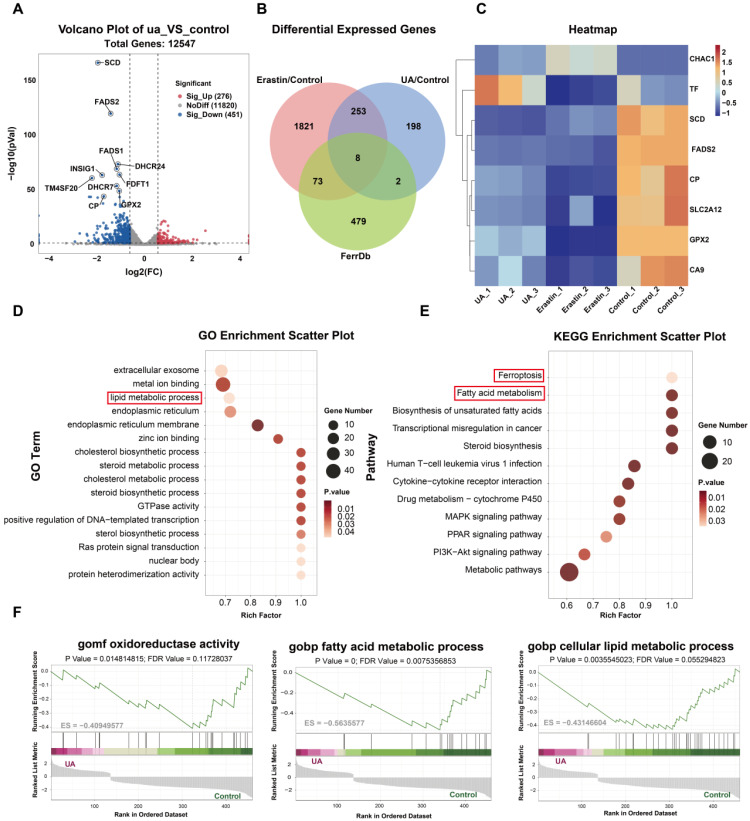
mRNA sequence analyses. (A)Volcano plot showed differentially expressed genes between the UA and control (DMSO) groups. (B)Venn diagram illustrated the differentially expressed genes of UA VS Control, Erastin VS Control, and FerrDb database. (C)Hierarchical clustering heatmap of 8 common genes. (D)GO analysis. (E)KEGG analysis. (F)GSEA results of C5 reference gene (GO gene sets) set of UA VS control differential genes.

**Figure 5:**
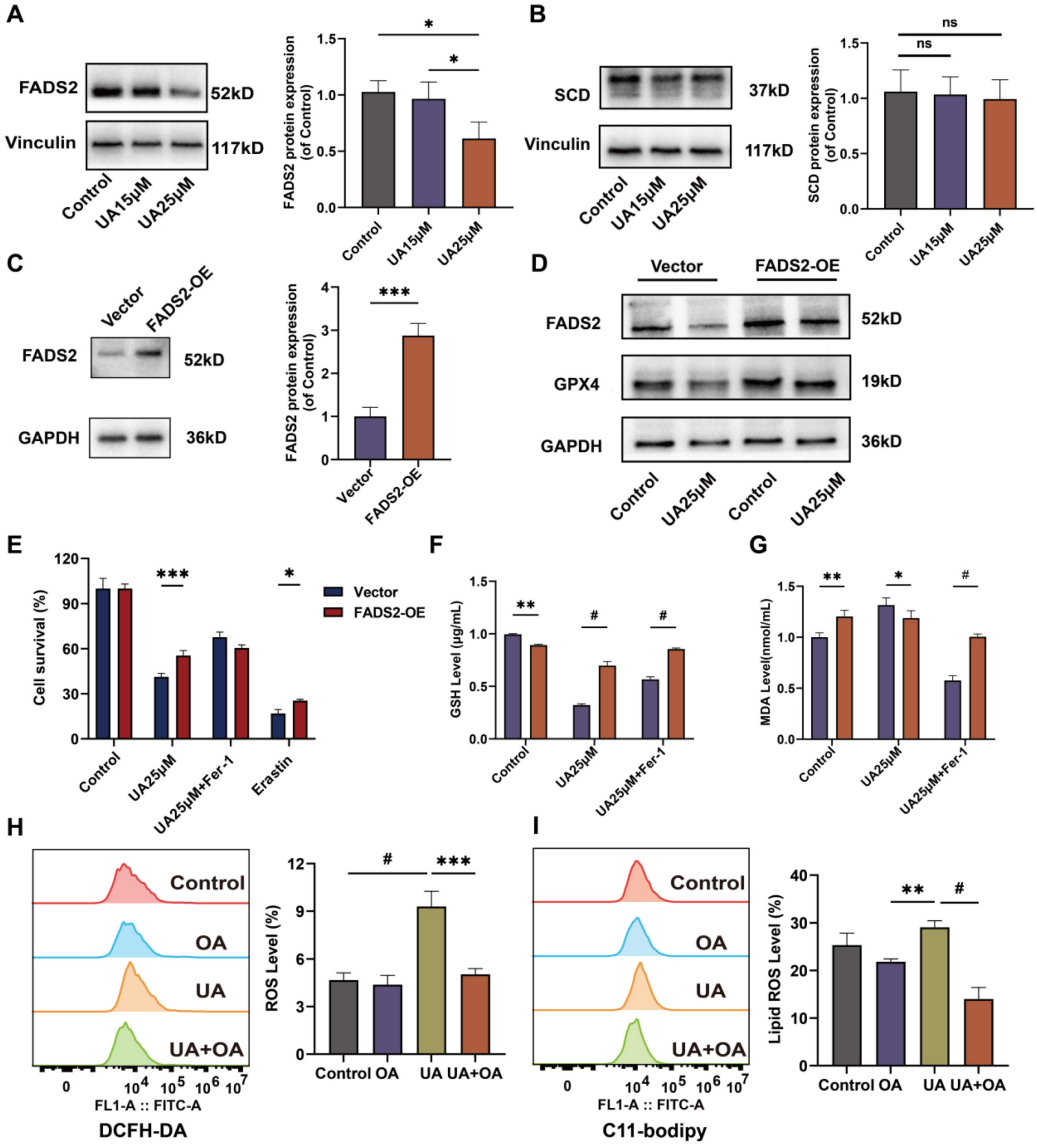
Overexpression of FADS2 renders A549 cells resistant to ferroptosis after UA treatment. (A)Western blot analysis of FADS2 and (B)SCD protein expression in UA-treated A549 cells. (C)FADS2 overexpression efficiency via Western blot analysis. (D) Changes in FADS2 and GPX4 protein levels after UA treatment of FADS2-OE A549 cell as compared to Vector. (E)FADS2-OE A549 and Vector A549 were treated with DMSO, UA (25μM), UA+Fer-1, Erastin(20μM) for 24h and cell viability was detected by CCK-8 assay. (F)Changes in GSH levels in FADS2-OE and Vector after UA and UA+Fer-1 for 24h. (G)Changes in MDA levels (histograms grouped as Fig 5 E). (H)DCFH-DA probe detected ROS changes after 24H of A549 after DMSO, OA (100μM), UA (25μM), and OA+UA combined were applied. (I)C11-Bodipy detected lipid ROS changes after the same treatments.

To further investigate the potential molecular mechanism of UA-induced ferroptosis in lung cancer cells, we performed mRNA sequencing and analyzed the changes of transcriptome genes in A549. We established three experimental groups: UA (25μM), Erastin (20μM used as a positive control for ferroptosis), and Control (DMSO). |Log_2_ fold change ≥ 1|, *P* value < 0.05, were used as the screening standards. The volcano plot shows the gene expression differences between the UA/Control groups, and genes with significant differences were annotated (Figure 4A). The functions of differentially expressed genes (DEGs) between the UA/Control groups were further explained using Kyoto Encyclopedia of Genes and Genomes (KEGG) pathway annotation analysis. We found that the differential genes were highly enriched mainly in pathways associated with ferroptosis and lipid metabolism (Figure 4D, E). To further validate ferroptosis-specific candidates, we compared the DEGs of UA VS Control, Erastin VS Control, and ferroptosis-related genes in the FerrDb database (Database URL: http://www.zhounan.org/ferrdb). The Venn plot showed 8 common genes (Figure 4B), which were displayed in the hierarchical clustering heatmap (Figure 4C). Furthermore, gene set enrichment analysis (GSEA) showed that UA treatment-induced gene expression regulated fatty acid synthesis, fatty acid metabolism, and redox pathways (Figure 4F). Taken above, The RNA-seq analysis revealed that DEGs in the UA group, compared to the Control group, were significantly enriched in lipid metabolism and ferroptosis pathways. This suggests that lipid metabolism-related processes may play a crucial role in the molecular mechanism through which UA induces ferroptosis in NSCLC. Consequently, we proceeded to investigate the biological processes associated with the lipid metabolism-related genes SCD and FADS2. To further verify the changes of these two genes at the protein level, the western blot assay was carried out and the results indicated that FADS2 protein level decreased significantly (Figure 5A), while SCD displayed a slight but not statistically significant decreasing tendency (Figure 5B).

### Ectopic overexpression of FADS2 renders A549 resistant to ferroptosis upon UA treatment

We transduced A549 with the FADS2-over-expressing plasmid via lentiviral transduction and puromycin selection to create A549 FADS2-OE cells. The efficiency of overexpression was detected by fluorescence observation (Figure S2) and western blot experiments (Figure 5C) The inhibitory effect of UA on cell viability was restored by overexpression of FADS2 (Figure 5E). Western blot results demonstrated that GPX4 expression was increased after overexpression of FADS2, acting similarly to Fer-1 treatment. Even under UA treatment, the decrease in GPX4 was not significant (Figure 5D). Furthermore, UA treatment significantly suppressed the level of GSH decline and the level of MDA elevation in FADS2-OE as compared with the Vector (Figure 5F, G), which had similar effects as Fer-1 treatment. To better illustrate the effect of FADS2 on ferroptosis, we modeled the environment of exogenous monounsaturated fatty acid (MUFA) by adding in Oleic Acid 25 and discovered that changes in ROS. The results revealed that lipid ROS were offset in the group with UA combined with OA compared to UA alone (Figure 5H, I). All the results suggested that overexpression of FADS2 could counteract the effect of UA on ferroptosis in NSCLC.

### *In vitro* studies confirmed the tumor-suppressing role of UA in NSCLC

**Figure 6:**
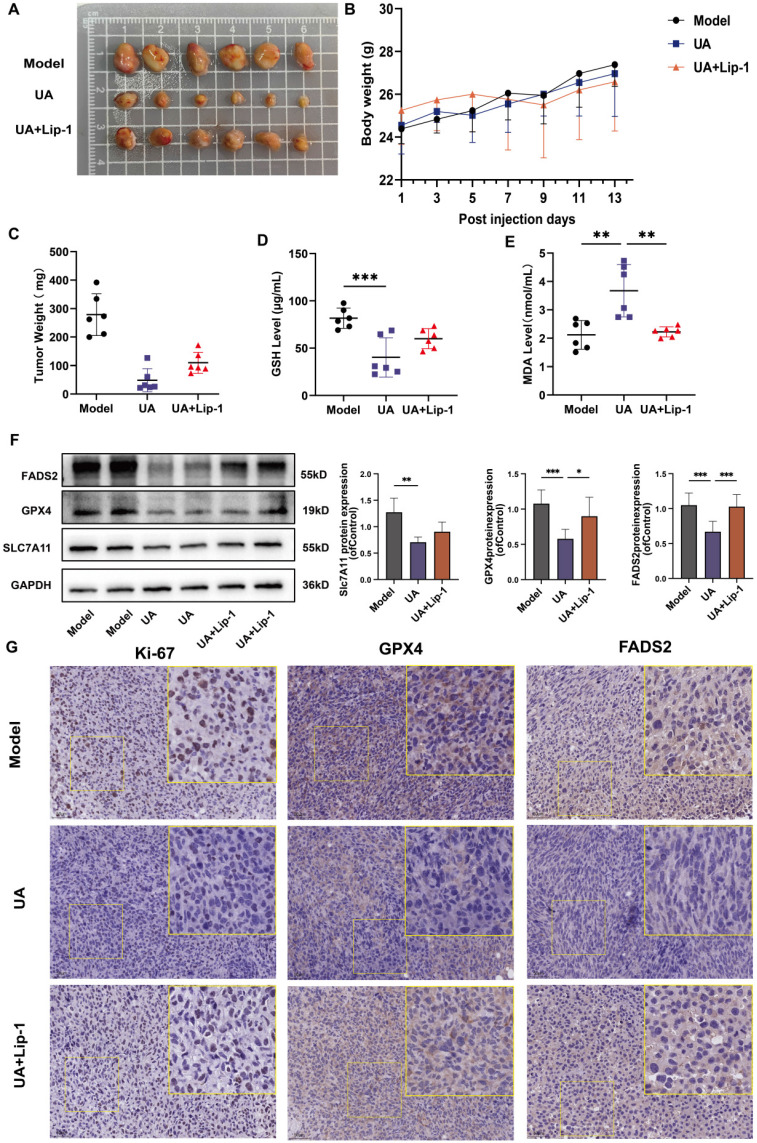
Effect of UA on subcutaneous xenograft tumors *in vivo*. (A)Tumor photo of each group. (B)Body weight. (C)Tumor weights (Data were plotted as mean ± SD, n = 6). (D)Serum levels of GSH and (E) MDA in each group of mice (n = 6). (F) GPX4, SLC7A11 and FADS2 protein expression levels in tumor tissues among different groups. The quantitative results of three replicate Western blot experiments. (G) Microscopic images of Ki-67 staining, and immunohistochemistry of GPX4 and FADS2 expression in different treated tumor tissues. (Scale bar, 50 μm).

We used a subcutaneous transplantation tumor model in BALB/c nude mice, which were given saline, UA (25 mg/kg), and a combination of UA with Lip-1 (10 mg/kg) treatments, respectively (Figure 6A). The mice's body weight was monitored while they were given the drug. There were no statistically significant differences between the groups, showing that the drug had no significant negative impact on the animals' health (Figure 6B). The weight of the tumors was significantly lower in the UA-treated group than in the other treatment groups (Figure 6C). This suggests that the killing effect of UA on tumor cells is achieved in part through the ferroptosis pathway, which can be inhibited by the ferroptosis inhibitor Lip-1. We tested GSH and MDA using serum from each group and showed a significant decrease in GSH levels (Figure 6D) and a clear increase in MDA levels (Figure 5E) in the UA group compared to the model group. However, this effect was partially mitigated by the Lip-1 combination treatment. The western blot results of protein extraction after tumor milling also indicated that the expression of GPX4, SLC7A11, and FADS2 proteins was decreased in the UA group (Figure 6F). Experimental analyses were conducted on HE staining (Figure S3), Ki-67 staining, and tumor immunohistochemistry. The Images and Bar graph (Figure S4) indicated a notable decrease in the proportion of Ki67 positivity, GPX4, and FADS2 levels in the UA group compared to the model or Lip group (Figure 6G). Taken together, these results further demonstrate that UA induces ferroptosis in animal models, disrupts redox homeostasis, and inhibits FADS2 expression.

## Discussion

The identification of ferroptosis has presented various promising opportunities for the therapeutic intervention of lung cancer. The established pharmaceuticals, including sorafenib, statins, imatinib, and Herbal Extracts artemisinin, as well as ionizing radiation and cytokines have exhibited the capacity to induce ferroptosis 26-28. Additionally, an expanding repertoire of compounds or nanocarriers, like Janus nanoparticles 29, have been discovered, which selectively target and induce ferroptosis. Thereby facilitating the investigation of targeted therapeutic approaches for lung cancer utilizing ferroptosis.

The previous study has unveiled that the co-administration of ursolic acid and sorafenib exhibits a synergistic impact by upregulating ROS levels and downregulating the expression of SLC7A11 in gastric cancer and colon cancer cells 20. It has also been shown that UA possesses the capability to inhibit the expression of hypoxia-inducible factor-1 alpha through a reduction in GSH levels within NSCLC, thereby increasing the sensitivity of radiotherapy 30. In this study, we demonstrate that UA inhibits NSCLC cell proliferation and tumor growth in the A549 xenograft tumor mouse model by inducing ferroptosis. UA exerts its ferroptosis effects by targeting the SLC7A11/GSH/GPX4 axis and the fatty acid metabolism-related protein FADS2.

The SLC7A11/GSH/GPX4 axis serves as a crucial intracellular antioxidant signaling pathway. SLC7A11 is a light chain subunit part of the System Xc^-^, which functions as a transporter protein located in the cell membrane. Cancer cells heavily rely on the SLC7A11 system to facilitate the transportation of cystine from the extracellular environment into the cell 31. The proliferation of tumors is contingent upon the nutrient substance (like cystine) to fulfill augmented biosynthetic and bioenergetic requirements while preserving redox equilibrium 32. Cystine is converted into cysteine within the cytoplasm through the reduction reaction of NADPH, ultimately promoting the synthesis of glutathione and GPX4 protein 33. GPX4, a member of the GPX enzyme family, is a crucial antioxidant enzyme within the cell responsible for converting lipid peroxides into lipid alcohols, thereby playing a significant role in ferroptosis^35^. Our research shows that UA serves as a disruptive agent, akin to the "Golden Apple of Discord," disturbing the dynamic redox equilibrium. The impact of UA is mediated through the suppression of SLC7A11 protein expression, resulting in reduced glutathione production and decreased GPX4 protein expression, thus preventing cells from inhibiting lipid peroxidation. The findings from fluorograms and flow analysis of Lipid ROS, as well as the measurement of lipid peroxidation substrate MDA, further support the presence of redox imbalance. Similar results were observed in animal experiments. Collectively, the evidence strongly suggests that UA induces a cellular shift towards an oxidized state, potentially induced by oxygen radicals or lipid peroxidation, thereby creating a conducive environment for the development of ferroptosis.

Based on the DEGs between the UA and Control groups of transcriptome sequencing, and the biological processes of GO and KEGG enrichment analysis, we discovered DEGs enriched in lipid metabolism.

SCD and FADS2 are pivotal enzymes within the fatty acid desaturation pathway and are markedly expressed in various cancer types. During *de novo* fatty acid synthesis, SCD facilitates the introduction of a double bond between the Δ9 and Δ10 positions in saturated fatty acids, specifically stearic acid (C18:0) and palmitic acid (C16:0), resulting in the formation of oleic acid (C18:1) and palmitoleic acid (C16:1), respectively 34,35. FADS2 is responsible for the conversion of linoleic acid into long-chain polyunsaturated fatty acids by encoding the delta-6 desaturase. Recent studies have also demonstrated that FADS2 can desaturate palmitate, producing sapienate with a double bond at the Δ6 position 36. In recent years, research has revealed a close relationship between unsaturated fatty acid synthesis and ferroptosis. Memorial Sloan Kettering Cancer Center has made the initial discovery that cancer cells harboring mutations in the PI3K-AKT signaling pathway have been shown to develop resistance to ferroptosis, attributed to the upregulation of the SREBP1/SCD protein complex, which governs fatty acid synthesis 37. Subsequent studies have reported that aspirin effectively diminishes SCD-mediated MUFA production and induces ferroptosis in colorectal cancer cells 38. Furthermore, CB1 has been found to regulate ferroptosis in triple-negative breast cancer cells by modulating SCD and FADS2 dependent fatty acid metabolism 7. Based on the available evidence and transcriptomic data, our research sought to explore the potential correlation between the observed changes in the lipid-related gene FADS2 and the enhancement of ferroptosis by UA in NSCLC. After the western blot assay, we found that FADS2 protein expression was significantly reduced after UA treatment on A549 cells. Upon investigation of FADS2-OE A549, it was found that overexpression of FADS2 enhanced cell viability after ua action, increased expression of GPX4 protein and restored degree of changes in GSH and MDA compared to the vector. These findings suggest A549 cell was resistant to UA-induced ferroptosis after FADS2 overexpression.

Based on the analysis of the TCGA dataset, the expression level of the FADS2 gene demonstrated a significant association with the survival outcomes of lung cancer patients. Specifically, patients in the low-expression FADS2 gene group exhibited longer overall survival (OS) and disease-specific survival (DSS) compared to those in the high-expression group (Figure S5). This observation aligns with the role of FADS2 in the prognosis of other malignancies 39. FADS2 was found to be highly expressed in a subset of triple-negative breast cancer patients characterized by poorer prognoses 40; furthermore, donafenib was shown to downregulate FADS2, resulting in synergistic inhibition of hepatocellular carcinoma in both *in vitro* and *in vivo* models 41. Genetic and metabolomic analyses have identified the FADS locus, which includes FADS1 and FADS2, as a critical regulatory site for the formation of biologically significant lipids. Variants within this gene are strongly correlated with the levels of long-chain polyunsaturated fatty acids (LC-PUFAs) 42. Cancer cells often exploit altered lipid biosynthesis and desaturation pathways to support rapid growth and survival, and lipid synthesis also influences the sensitivity of cancer cells to ferroptosis. In this context, FADS2 emerges as a promising target for therapeutic intervention in cancer treatment.

It is noteworthy that FADS2 is frequently identified in the literature as a key enzyme involved in the synthesis of polyunsaturated fatty acids, thereby enhancing susceptibility to ferroptosis. The non-canonical desaturation of oleate to Mead acid and other highly unsaturated fatty acids, mediated by FADS2, facilitates the ferroptosis pathway, which in turn attenuates Hepatitis C viral replication 43. In triple-negative breast cancer, TNBC with high FADS1/2 expression were found to be highly sensitive to ferroptosis and inhibition of FADS1/2 expression decreased the ratio of polyunsaturated fatty acids and inhibited ferroptosis 40. However, based on experimental findings and a comprehensive literature review, we propose the hypothesis that this phenomenon may be attributed to a distinct subpopulation of cancer cells, specifically lung cancer cells, which may adopt an alternative desaturation pathway for fatty acid metabolism. Certain cancer types, including lung and liver cancers, circumvent the SCD-dependent fatty acid desaturation pathway during proliferation to produce monounsaturated fatty acid. Lung cancer, in particular, exhibits insensitivity to the inhibitory effects of SCD, favoring enhanced activity of FADS2. Analyses of clinical samples have also demonstrated elevated levels of FADS2 circular RNA in lung cancer tissues, which are associated with reduced overall survival in lung cancer patients 36. Kohsei's research demonstrated that FADS2 was significantly expressed in clinical samples of cholangiocarcinoma, while it was not detected in stromal cells. The application of a FADS2 inhibitor resulted in a reduction of GPX4 protein expression levels and an elevation of ferroptosis markers 44. Further investigations revealed that the inhibition of SCD1/FADS2 in ascites-derived ovarian cancer cells led to the downregulation of GPX4 and a decrease in GSH levels, which subsequently induced lipid peroxidation and mitochondrial dysfunction, triggered cellular ferroptosis, and heightened sensitivity to cisplatin 45. These findings underscore the intricate role of FADS2 in modulating ferroptosis.

We hypothesize that UA, as a potential inhibitor of SCD and FADS2, suppresses the synthesis of monounsaturated fatty acids (MUFAs), leading to a relative increase in polyunsaturated fatty acids (PUFAs) and thereby enhancing susceptibility to ferroptosis. In our study, co-administration of UA with exogenous MUFAs, specifically OA, resulted in a reduction of UA-induced ROS and lipid ROS levels. Further research is necessary to elucidate the precise impact of lipid alterations on ferroptosis susceptibility in the presence of UA.

## Conclusion

In conclusion, our study illustrated that Ursolic Acid exerts a suppressive impact on non-small cell lung cancer proliferation by promoting ferroptosis, as evidenced by findings from cellular and animal trials. Remarkably, UA enhances susceptibility to ferroptosis by impeding the SLC7A11/GSH/GPX4 Axis and suppressing the fatty acid desaturase FADS2, suggesting promising prospects for UA's clinical utility in cancer treatment.

Animal experiments abide by the requirements of the National Institutes of Health guide for the care and use of Laboratory animals (NIH Publications No. 8023, revised 1978).

## Supplementary Material

Supplementary figures and tables.

## Figures and Tables

**Figure 2:**
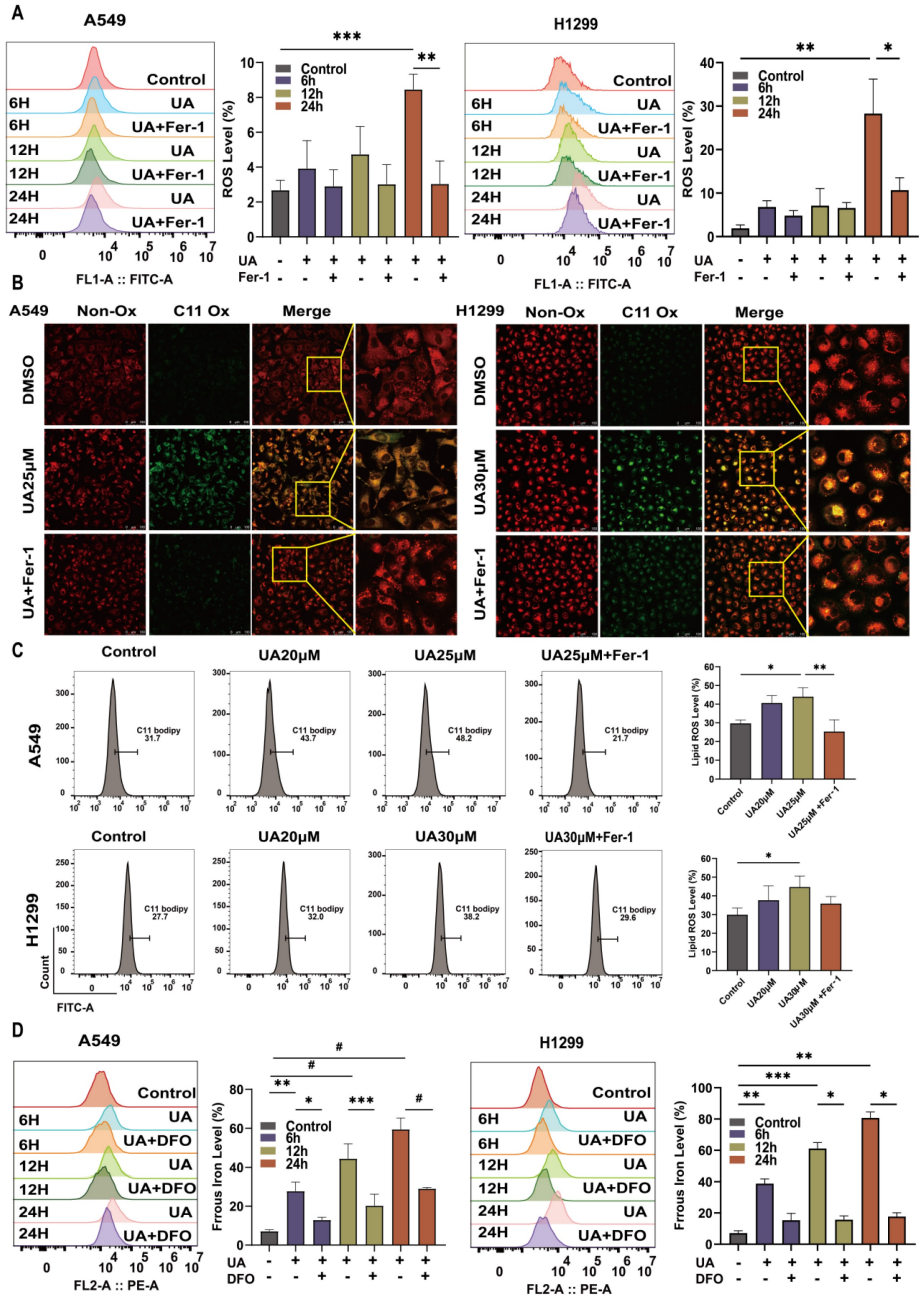
UA increases ROS\lipid ROS\ferrous iron production in NSCLC. (A)Flow cytometry was performed to detect the changes of A549\H1299 ROS in UA, UA+Fer-1,and DMSO treatment at different times (0,6,12,24h). (B)Confocal images of lipid peroxidation by UA, UA+Fer-1, and DMSO action for 24h. The red color indicates non-oxidized state while the green color indicates oxidized state. Scale bar,100 μm. (C)Changes in lipid peroxidation were detected by flow cytometry. (D) FerroOrange probe to detect changes in ferrous iron (Bar graphs represent the proportion of changes)
