# Supplementary material for: Ursolic Acid induces ferroptosis by affecting redox balance and FADS2-mediated unsaturated fatty acid synthesis in Non-Small Cell Lung Cancer
Source: J Cancer. 2025 May 27;16(8):2553–66. doi: 10.7150/jca.105863 (PMC12170995; doi:10.7150/jca.105863)
Supplement: Supplementary file 1 — Supplementary figures and tables. [file jcav16p2553s1.pdf]

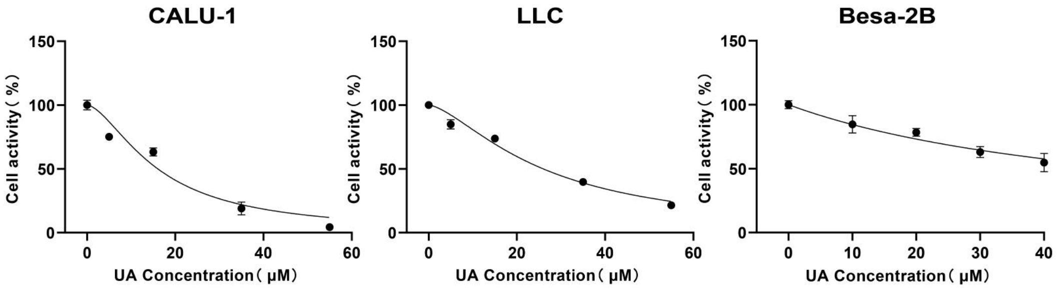

Figure S1: The cell activity of Calu-1, LLC, Besa-2B were determined by the CCK-8 method after 24h of UA treatment.

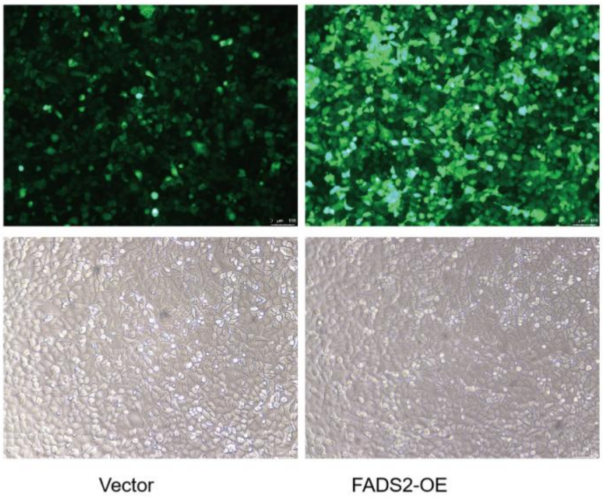

Figure S2: The efficiency of overexpression was detected by fluorescence observation

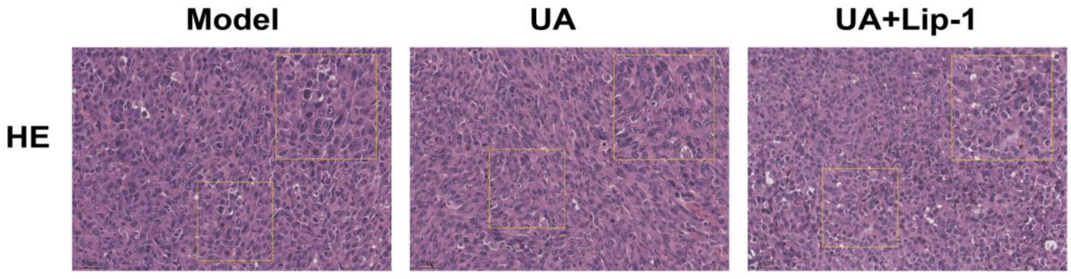

Figure S3: He staining of tumors in various groups

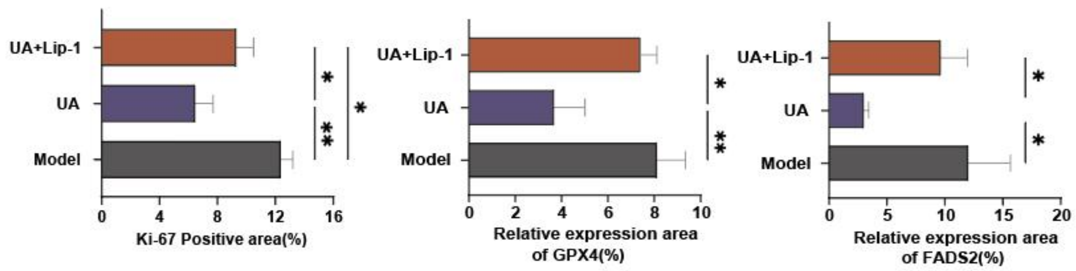

Figure S4:Bar graph of IHC positive area statistics for each group

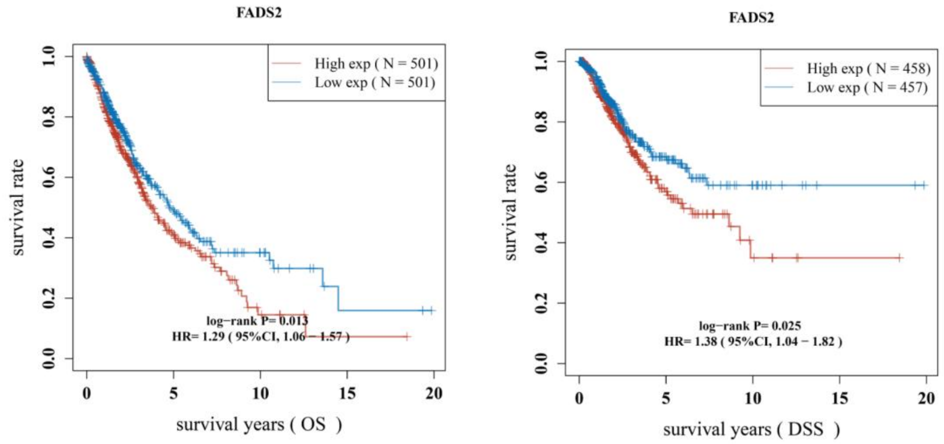

Figure S5 : Kaplan-Meier curve analysis of OS and DSS in high and low FADS2 expression groups in lung cancer
